# The comparison of CHCA solvent compositions for improving LC-MALDI performance and its application to study the impact of aflatoxin B1 on the liver proteome of diabetes mellitus type 1 mice

**DOI:** 10.1371/journal.pone.0181423

**Published:** 2017-07-24

**Authors:** Fuu-Jen Tsai, Shih-Yin Chen, Yu-Ching Liu, Hsin-Yi Liao, Chao-Jung Chen

**Affiliations:** 1 Graduate Institute of Chinese Medical Science, College of Chinese Medicine, China Medical University, Taichung, Taiwan; 2 Genetic Center, Department of Medical Research, China Medical University Hospital, Taichung, Taiwan; 3 Proteomics Core Laboratory, Department of Medical Research, China Medical University Hospital, Taichung, Taiwan; 4 Graduate Institute of Integrated Medicine, College of Chinese Medicine, China Medical University, Taichung, Taiwan; National Research Council of Italy, ITALY

## Abstract

In nanoflow liquid chromatography-matrix-assisted laser desorption/ionization tandem time-of-flight (nanoLC-MALDI-TOF/TOF) approaches, it is critical to directly apply small amounts of the sample elutes on the sample target using a nanoLC system due to its low flow rate of 200 ~ 300 nl/min. It is recommended to apply a sheath liquid containing a matrix with a several μL/min flow rate at the end of the nanoLC column to ensure a larger co-eluted droplet for more reproducible sample spotting and avoid the laborious task of post-manual matrix spotting. In this study, to achieve a better nanoLC-MALDI performance on sample spotting, we first compared α-Cyano-4-hydroxycinnamic acid (CHCA) solvent composition for efficiently concentrating nanoLC elutes on an anchor chip. The solvent composition of isopropanol (IPA): acetonitrile (ACN):acetone:0.1% Trifluoroacetic acid (TFA) (2:7:7:2) provided strong and homogeneous signals with higher peptide ion yields than the other solvent compositions. Then, nanoLC-MALDI-TOF/TOF was applied to study the impact of aflatoxin B1 on the liver proteome from diabetes mellitus type 1 mice. Aflatoxin B1 (AFB1), produced by *Aspergillus flavus and Aspergillus parasiticus* is a carcinogen and a known causative agent of liver cancer. To evaluate the effects of long-term exposure to AFB1 on type 1 diabetes mellitus (TIDM), the livers of T1DM control mice and mice treated with AFB1 were analyzed using isotope-coded protein labeling (ICPL)-based quantitative proteomics. Our results showed that gluconeogenesis, lipid, and oxidative phosphorylation mechanisms, normally elevated in T1DM, were disordered following AFB1 treatment. In addition, major urinary protein 1 (MUP1), an indicator of increased insulin sensitivity, was significantly decreased in the T1DM/AFB1 group and may have resulted in higher blood glucose levels compared to the T1DM group. These results indicate that T1DM patients should avoid the AFB1 intake, as they could lead to increased blood glucose levels and disorders of energy-producing mechanisms.

## Introduction

Diabetes mellitus (DM) is a metabolic disease involving chronic hyperglycemia. Type 1 diabetes results from the destruction of pancreatic β-cells, which are responsible for insulin secretion. Complications of type 1 diabetes mellitus (T1DM) include altered energy and glucose metabolism, cardiovascular disease, stroke, neuropathies, nephropathy [1], and retinopathy [2]. Liver disease, such as nonalcoholic steatohepatitis, is also a common complication found in T1DM patients. The liver is one of the principal organs involved in the regulation of carbohydrate metabolism. Normal liver function is essential to modulate blood glucose levels when necessary and supply glucose to organs.

Aflatoxin B1 (AFB1) is a type of natural mycotoxin produced by *Aspergillus flavus* and *Aspergillus parasiticus*, and is present as a contaminant in various foods, such as meat, milk, and eggs [3]. The liver, a major organ of metabolism, is the principal target organ for AFB1. Because T1DM patients may have dietary exposure to AFB1, it is important to reveal the possible impact of AFB1 on liver proteome of T1DM.

Proteomics studies have long been used for protein identification and description of various biochemical processes, pathways, and mechanisms in both normal and abnormal physiological states [4]. Isotope-Coded Protein Labeling (ICPL) is one of several chemical labeling strategies for the identification and relative quantitation of complex proteomes [4–6]. In the analysis of labeled-peptides, nanoflow liquid chromatography-mass spectrometry (nanoLC-MS) with nano-electrospray ionization (nanoESI) source is widely adapted due to the high sensitivity and complete automation of data acquisition. In nanoLC-ESI MS analysis, tandem mass spectrometry (MS/MS) acquisitions are triggered by “data-dependent” analysis of the intense peaks in the MS spectrum. However, the acquired numbers of MS/MS spectra are usually limited by chromatography peak width, data-dependent cycle time and MS scan rates. In addition, MS/MS acquisitions are usually triggered at positions off the apex of the peak, resulting in a loss in sensitivity. To solve the above limitations of nanoLC-ESI-MS/MS, an attractive approach has been proposed for acquiring MS/MS spectra by using nanoLC coupled with MALDI-TOF/TOF (nanoLC-MALDI-TOF/TOF) [7–10]. Compared to nanoLC-ESI/MS/MS, the off-line MALDI interfaces allow eluents from the LC column to be deposited and archived on the MALDI target for MS/MS analysis with optimum sensitivity at the maximum of the chromatographic peak. In addition, tryptic peptide ions in MALDI are predominately singly charged ions, which results in less complex MS spectra and minimizes redundant acquisitions compared to nanoLC-ESI-MS/MS.

An MALDI anchor chip, usually adopted in nanoLC-MALDI-TOF/TOF approaches, is used to pre-concentrate the LC elutes on the sample well [11–13] and locate samples in the middle of the sample well for automatic acquisition of MS and MS/MS spectra. However, it is critical to directly apply small amounts of the sample elutes on the sample target using a nanoLC system due to a nanoLC flow rate of 200 ~ 300 nl/min. A sheath liquid containing a matrix with a several μL/min flow rate has been recommended for application at the end of the nanoLC column to ensure a larger co-eluted droplet for more reproducible sample spotting and avoid the laborious task of post-manual matrix spotting. However, matrix solvent compositions can greatly influence sample/matrix heterogeneity and the pre-concentration factor on the anchor chip.

Therefore, in this study, we first compared several reported α-Cyano-4-hydroxycinnamic acid (CHCA) solvent composition for efficiently concentrating sample droplets on an anchor chip with higher peptide ion yields. The better CHCA solvent composition was applied to the ICPL-nanoLC-MALDI-TOF/TOF platform to obtain a more comprehensive picture of proteomic changes in the liver related to T1DM and to study the possible impact of an AFB1-contaminated diet on T1DM patients.

## 2. Materials and methods

### 2.1 MALDI-TOF and MALDI-TOF/TOF acquisition in matrix solution comparison experiments

All mass-analyses were performed on an Ultraflex III (Bruker Daltonics) equipped with a 200 Hz Smartbeam laser system. For matrix solvent comparison experiments, 10 MS spectra produced from one sample spot were combined for Mascot protein database search using peptide mass fingerprinting (PMF). For MS/MS results, MS/MS spectra were acquired using auto-execute automatic acquisition throughout the sample spot for obtaining peptide fragmented ion spectra. The protein search results from MS or MS/MS data were averaged from five replicated measurements (five sample spots of BSA tryptic peptides).

### 2.2 MALDI imaging

In order to observe the effect of matrix solvent composition on signal homogeneity, MALDI imaging approach was used for acquiring signals throughout the sample spots. Samples of bovine serum albumin (BSA) digests (5 fmol) were mixed with various matrix solutions and deposited on sample spots. After drying, the sample plate was scanned to obtain an image file. The file was then imported into Fleximaging 2.0 software (Bruker-Daltonics). The acquired data range was circled on the scanned image. The auto-execute function allows mass spectra to be automatically acquired within the circled rage. A data imaging file was finally generated through the conversion of the mass spectrum obtained for each pixel and association with its X and Y coordinates. Intensity scale 0–0.4 served to visualize larger intensity range, which allows lower intensity distinction by colors.

### 2.3 Animals and induction of diabetes

All experimental procedures were performed according to the NIH Guide for the Care and Use of Laboratory Animals, and all protocols were approved by the Institutional Animal Care and Use Committee of China Medical University, Taichung, Taiwan. Sixteen male C57BL/6 mice at 8 weeks of age were obtained from the National Laboratory Animal Center, Taiwan. Their ambient temperature was maintained at 25°C, and the animals were kept on an artificial 12-h light-dark cycle. The light period began at 7:00 A.M. Mice were provided with standard laboratory chow (Lab Diet 5001; PMI Nutrition International Inc., Brentwood, MO, USA) and water *ad libitum*.

All animals were set to adapt to the environment for 1 week after their arrival before the experiment started. All ten-weeks old mice were divided into two groups: the control group (n = 6) and streptozotocin (STZ)-injected group (n = 10). The mice in the STZ-injected group were injected with STZ (in citrate buffer, pH 4.5) (65 mg / kg / 7 days / 3 cycle), and the mice in the control group were injected with an equal volume of vehicle. The mice were considered to be diabetic if their fasting glucose levels were maintained at > 11.1 mM or > 200 mg/dl 48 h after injection of STZ as measured with AccuSoft (Hoffmann-La Roche) test strips. Three days after the first injection of STZ, four of the ten STZ-induced diabetic mice were treated with AFB1 (10 mg/kg of body weight) by intraperitoneal injection. AFB1 (Sigma Co, St. Louis, MO) was dissolved in tricaprylin (Sigma Co, St. Louis, MO) to a concentration of 0.2 mg/mL. There were three groups in the study: a control group with citrate buffer (WT) (n = 6), STZ-induced diabetic mice with DMSO (T1DM) (n = 6), and STZ-induced diabetic mice with AFB1 treatment (T1DM/AFB1) (n = 4). All mice were sacrificed by CO_2_ asphyxiation at 48 weeks. The blood was immediately collected by cardiac puncture, and liver tissues were harvested and frozen for proteome analysis.

### 2.4 Tissue homogenization and protein preparation

Frozen pieces of liver were weighed and then pulverized with a liquid nitrogen-chilled mortar and pestle. Liver tissue samples were homogenized with 3 volumes of ice cold phosphate buffer (10 mM, pH 7.0) containing 0.25 M sucrose, 1 mM Ethylenediaminetetraacetic acid (EDTA), 1 mM sodium azide, and 0.1 mM phenymethylsulfonylflouride. After centrifugation at 20,000 ×g for 30 min at 4°C, the supernatant containing proteins was collected. Protein concentrations were determined using a bicinchoninic acid protein assay (Pierce, Rockford, IL) and standard curves of BSA in appropriate buffers.

### 2.5 Isotope-coded protein and peptide labeling

For ICPL protein labeling, protein samples (40 μg) from each group from each group (control, T1DM, and T1DM/AFB1) were dissolved in 20 μl of 25 mM TEAB buffer (triethyl ammonium bicarbonate) and reduced with 0.5 μL of 0.2 M Tris (2-carboxyethyl) phosphine hydrochloride (TCEP) (final concentration of 5 mM) for 50 min at 60°C, followed by alkylation with 0.5 μL of 0.4 M iodoacetamide (IAA) (final concentration of 9.5 mM) for 30 min at 25°C in the dark. Then, 0.5 μL of stop solution 1 (N-acetyl-cysteine) was then added to the sample, followed by incubation for 15 min at 25°C to quench excess IAA. For ICPL protein labeling, the reduced and alkylated protein samples were labeled using 3 μL of ^12^C-Nic- (ICPL_0), ^2^D-Nic- (ICPL_4) and ^13^C^2^D-Nic-(ICPL_10) reagent solution (ICPL quadruplex-kit, Serva Electrophoresis GmbH, Heidelberg, Germany) for the respective sample. Because 3 μL of labeling reagents is sufficient to label 100 μg of total protein according to its instruction manual, the protein amount (40 μg) in this study can be completely labeled. The samples were incubated for 3 hours at 25°C with gentle vortexing. Labeling was then quenched by the addition of 2 μL of stop solution 2 (hydroxylamine), and all labeled protein samples were combined. Excess reagent was removed by precipitation with 150 μL of acetone at -20°C overnight. Sample pellets were resuspended with protein denaturing buffer followed by SDS-PAGE protein separation.

For ICPL peptide labeling, 40 μg of protein from each group (control, T1DM, and T1DM/AFB1) was reduced and alkylated as described earlier. The proteins were subsequently recovered through acetone precipitation, dissolved in 20 μl of 25 mM TEAB buffer (triethyl ammonium bicarbonate), 4 M urea, pH 8.5. After diluting the sample solution with 60 μl of 25 mM TEAB buffer to reduce the urea concentration to 1 M the sample solution was submitted to trypsin digestion for 12 h at 37°C (enzyme:substrate ratio of 1:25). The tryptic-digested peptides from each group were then labeled using 3 μL of 12C-Nic- (ICPL_0), 2D-Nic- (ICPL_4) and 13C2D-Nic-(ICPL_10) reagent solution and quenched by the addition of 2 μL of stop solution 2 (hydroxylamine). The ICPL-labeled peptides from the control, T1DM, and T1DM/AFB1 groups were pooled and purified with C18 solid phase extraction (Oasis, HLB, Waters, USA) and then fractionated with an strong cation exchange (SCX) column.

### 2.6 Fractionation of ICPL-labeled proteins by SDS-PAGE and In-gel digestion

ICPL labeled proteins were solubilized with 10% sodium dodecyl sulfate (SDS), and separated on 10% Tris-Glycine gel. Electrophoresis was performed at 75 V/gel and 110 V/gel for the stacking and resolving gels, respectively. After the separation, the gels were stained with Coomassie brilliant blue G250. The protein gels were evenly cutted into 18 gel bands for protein fractionation.

Excised gel bands from SDS-PAGE were cut into small pieces and washed with 25 mM ammonium bicarbonate (ABC) solution (pH 8.2) containing 50% ACN for 15 min. Gel pieces were dehydrated with 100% ACN, reduced by 10 mM DTT at 56°C for 15 min., alkylated with 55 mM IAA in the dark at room temperature for 20 min., then washed by 25 mM ABC (pH 8.2) containing 50% ACN for 10 min. Gels were digested with trypsin (1:50 trypsin to protein ratio in weight) at 37°C overnight. After digestion, tryptic peptides were extracted from the gel using 0.1% TFA in 50% ACN. Extracted tryptic protein digest solution was dried by centrifugal concentrator, followed by nanoLC-MALDI-TOF/TOF analysis.

### 2.7 Fractionation of ICPL-labeled peptides by SCX chromatography

For peptide fraction, ICPL-labeled peptides were mixed with buffer A (5 mM KH_2_PO_4_ and 25% (v/v) ACN, pH 3.0) and loaded onto a 2.1 x 200-mm polysulfoethyl A column containing 5-μm particles with a 200-μm pore size (PolyLC, Columbia, MD). The peptides were eluted at a flow rate of 200 μL/min with a gradient of 0–35% buffer B (5 mM KH_2_PO4, 350 mM KCl, and 25% (v/v) ACN, pH 3.0) for 35 min followed by a gradient of 35–100% buffer B for 10 min. The fractions were collected every 1 min. Each fraction was vacuum dried and then resuspended in 0.1% (v/v) TFA (40 μL) for further desalting and concentration using ZipTips (Millipore, Bedford, CA).

### 2.8 LC-MALDI TOF/TOF for ICPL-labeled peptides

LC was performed using an Ultimate 3000 nanoLC system (Dionex, CA, USA). Samples were loaded into a trap column (Acclaim PepMap 100, C18, 5 μm, 100A, 300 μm i.d. x 5 mm, Dionex) followed by a 50-min gradient elution from 10% ACN/0.1% FA to 40%ACN/0.1% FA through an LC separation column (Atlantis dC18, 3 μm, 75 μm i.d. x 150 mm, Waters). The LC system was connected in-line with a Proteineer FC robotic fraction collector (Bruker Daltonics, Germany). The LC eluents were deposited onto an Anchorchip MALDI plate (800 μm anchor size, Bruker Daltonics) by mixing with the sheath flow of α-cyano-4-hydroxycinnamic acid (CHCA) (1 mg/mL) dissolved in IPA/ACN/acetone/0.1%TFA (2:7:7:2) solution at the LC column outlet at 2 μL/min using an internal syringe pump.

MALDI-MS analysis was performed on a MALDI-TOF/TOF (Ultraflex III, Bruker Daltonics) equipped with a 200 Hz Smartbeam laser system. Auto-execute automatic acquisition of mass spectra of nanoLC-fractions was controlled using WARP-LC software (version 1.2, Bruker Daltonics) interfaced with FlexControl. Data were acquired in WARP-LC method with automated acquisition of parent ions and selection of up to 15 ICPL-labeled peptide precursor ions with a signal-to-noise ratio (S/N) > 20 per target spot for subsequent MS/MS analysis. Laser shots for MS and MS/MS acquisition were 800 and 2400, respectively. MS peaks appearing in more than 50% of spectra were defined as background.

External calibration was performed automatically on Anchorchip calibration spots, on which Peptide Calibration Standard II (Bruker Daltonics) was spotted.

Auto execute data files from WARP-LC containing combined peptide MS and MS/MS spectra were automatically processed in FlexAnalysis 3.0 (Bruker Daltonics) and then imported into BioTools 3.0 (Bruker Daltonics) for a MASCOT protein search against the NCBI database with the following parameter settings: taxonomy-mice; fixed modification- carbamidomethylation (C), ICPL (K and N term); variable modification-oxidation (M, H, W); and 100 ppm MS tolerance, 0.3 Da MS/MS tolerance, minimum Mascot protein score > 50, and peptide score > 25. False discovery rates (FDRs) on MASCOT were estimated to be <0.3%. Only proteins identified with at least two peptides were taken into account. The relative quantitation of ICPL-labeled peptides based on signal intensities of MS spectra was performed automatically in conjunction with WARP-LC and examined manually in WARP-LC ProteinBrowser (Bruker Daltonics).

## 3. Results

### 3.1 Comparison of CHCA matrix solutions by sample homogeneity

Due to the low flow rate of 200 ~ 300 nl/min in nanoLC system, it is difficult to directly apply small amounts of the sample elutes on the sample target. A sheath liquid containing a matrix with a several μL/min flow rate at the end of the nanoLC column was usually used to ensure a larger co-eluted droplet for more reproducible sample spotting and avoid the laborious task of post-manual matrix spotting. However, matrix solvent compositions can greatly influence sample/matrix heterogeneity and the pre-concentration factor on the MALDI anchor chip. To discover a suitable CHCA matrix solution for nanoLC-MALDI analysis, the following reported matrix solution were tested.

(A) EtOH:Acetone (2:1) (recommend formula from Anchorchip user instruction) (B) 100%Acetone[14] (C) 50%ACN, 0.1%TFA[15, 16] (D) IPA: H2O:FA(2:3:1)[17, 18] (E) EtOH:ACN:H2O (60:36:4)[19] (F) IPA:ACN:Acetone:0.1%TFA (2:7:7:2)[20] (G) IPA:ACN:Acetone:0.1%TFA (2:7:7:2) with nitrocellulose (1 mg/mL) addition.[20]

**Fig 1:**
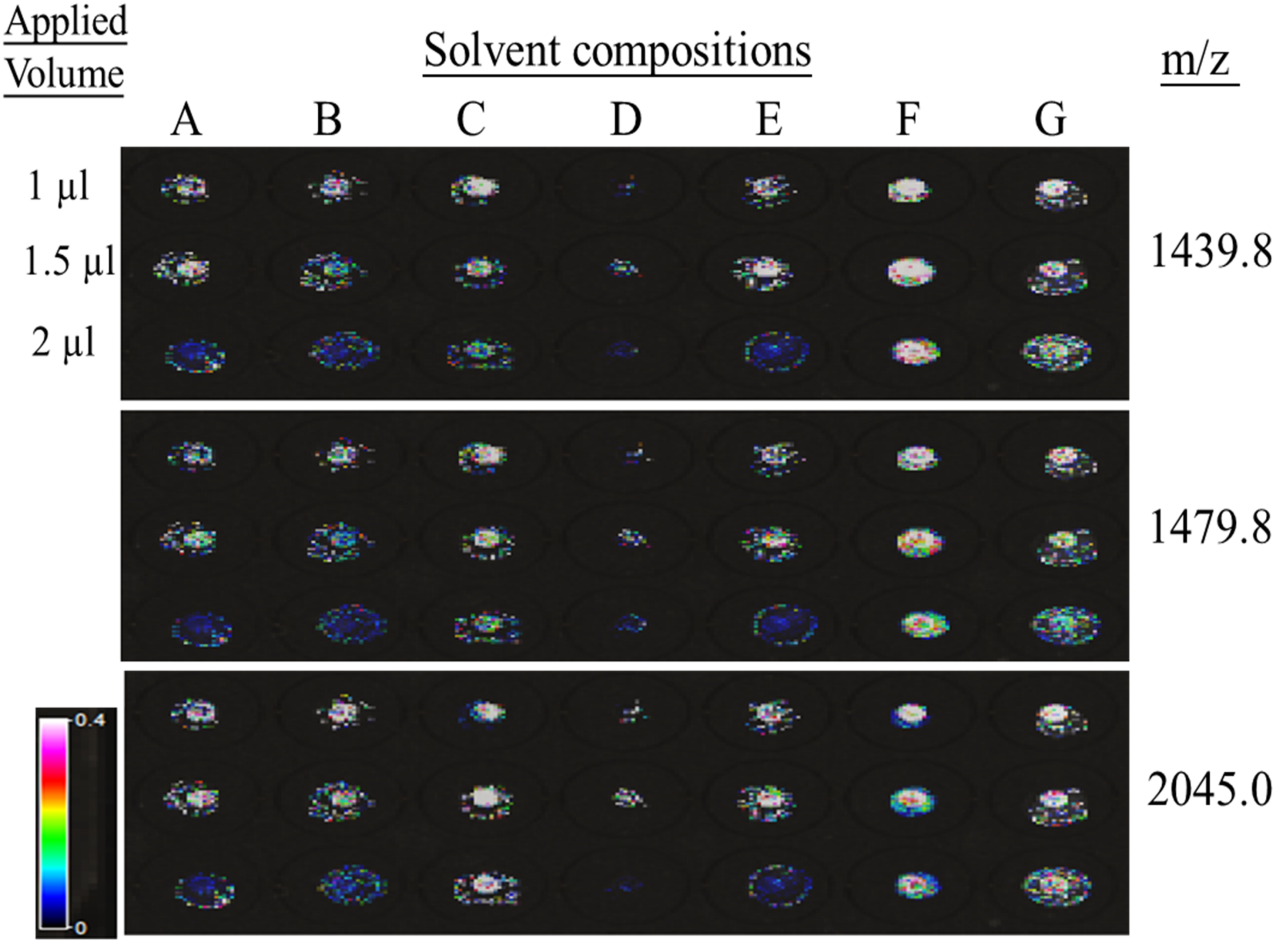
MALDI imaging acquisition of representative tryptic BSA peptides with various CHCA solvent compositions. CHCA (1mg/mL) was dissolved in (A) EtOH:Acetone (2:1) (B) 100%Acetone (C) 50%ACN, 0.1%TFA (D) IPA: H2O:FA(2:3:1) (E) EtOH:ACN:H2O (60:36:4) (F) IPA:ACN:Acetone:0.1%TFA (2:7:7:2) (G) IPA:ACN:Acetone:0.1%TFA (2:7:7:2) with nitrocellulose (1 mg/mL) addition.

In order to study the effect of matrix solvent composition on sample homogeneity, the MALDI image strategy was used for acquiring signals throughout the sample spots. As shown in Fig 1, various matrix solutions with BSA digests having 1 μL, 1.5 μL, and 2 μL total volumes were spotted on the anchor sample target. White color spots represent the strongest signals, and black color spots represent the weakest MS signals. Three representative peaks of BSA tryptic digests, 1439.8 m/z, 1479.8 m/z, and 2045.0 m/z, were used to evaluate sample homogeneity. Usually, a larger volume applied on an anchor will increase the sample spot area, reducing the concentration factor. However, this phenomenon was not observed with the F composition (IPA/ACN/Acetone/0.1%TFA, 2/7/7/2) because the F composition showed the strongest signals (white colors) of the three peaks in the anchor circle with a similar spot area and high sample homogeneity.

### 3.2 Comparison of CHCA matrix solutions by peptide yield and durability

**Fig 2:**
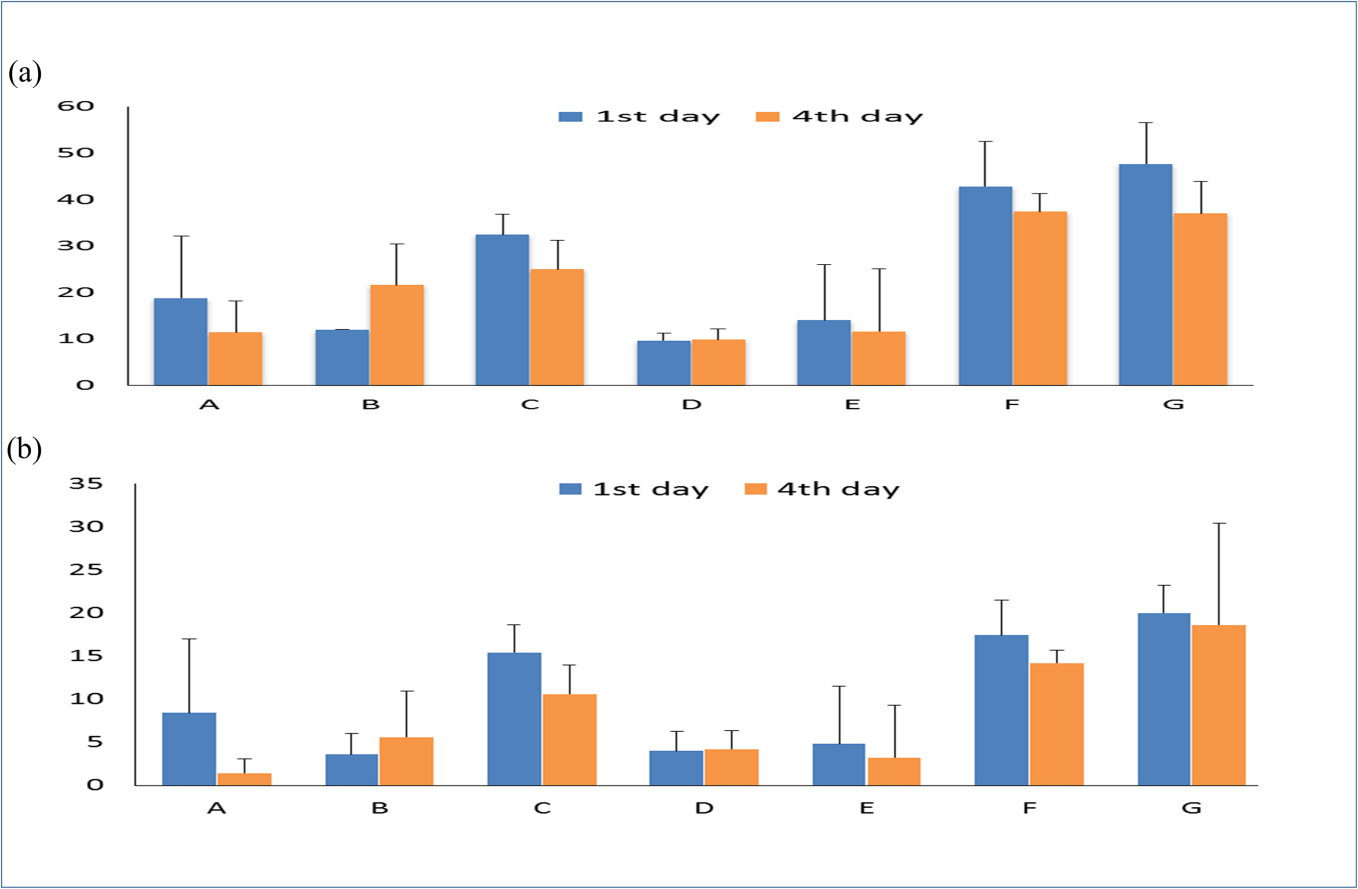
BSA sequence coverage comparison on different CHCA matrix solution. (a) PMF sequence coverage (%) and (b) MS/MS sequence coverage (%) with various matrix compositions. The sequence coverage was the average value of 5 replicated measurements.

In addition to sample homogeneity, improvement of the peptide yield and durability of the sample spots will also increase the protein sequence coverage of PMF and MS/MS acquisition. As shown in Fig 2, the F and G matrix compositions showed the best peptide yields (sequence coverage by PMF) and MS/MS durability values (sequence coverage by MS/MS) even after storage for 4 days. The G matrix composition consisted of IPA/ACN/acetone/0.1%TFA (2:7:7:2) with the addition of nitrocellulose and has been reported to have an improved peptide yield[20, 21]. Interestingly, we noticed that the F matrix composition, consisting of the same solvent composition as G but without the addition of nitrocellulose, led to a significantly improved peptide yield and MS/MS durability. Therefore, the organic solvent composition of IPA/ACN/acetone/0.1%TFA (2:7:7:2) is better than nitrocellulose to improve peptide detection performance. In addition, we also found that the addition of nitrocellulose in the sheath liquid might clog the outlet end of the nanoLC column, leading to unstable sample spotting on the MALDI target. Therefore, the F matrix composition was selected for the LC-MALDI platform to study the liver proteome of T1DM mice.

### 3.3 Proteome quantification by ICPL-nanoLC-MALDI-TOF

**Fig 3:**
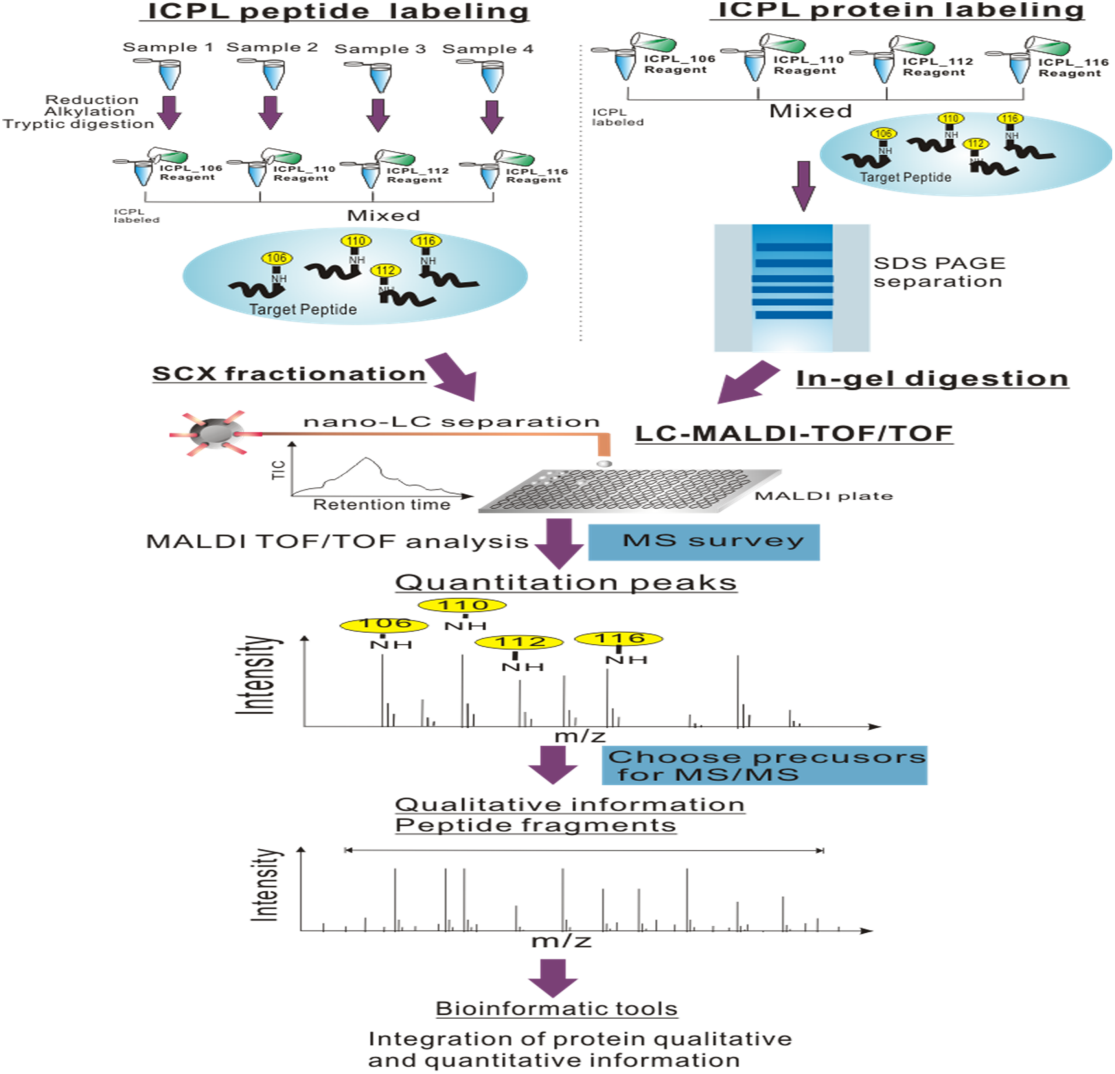
Flow chart of ICPL-peptide labeling- LC-MALDI and ICPL-protein labeling-LC-MALDI.

ICPL is a multiplexed comparative method for plural samples and is originally developed for labeling proteins rather than peptides [22]. The “post-digest ICPL” protocol, which labels peptides after digestion, was later performed by Leroy et al. [23] to increase the number of quantifiable peptides. In this study, we combined both protein and peptide labeling methods for a more comprehensive analysis; our experimental flow chart is shown in Fig 3. Proteins ratios differed by more than two standard deviations of 0.44 from the mean (1.03) were regarded as differentially expressed. Therefore, the statistical thresholds for protein differential expression were determined to be greater than 1.5 or less than 0.67.

**Fig 4:**
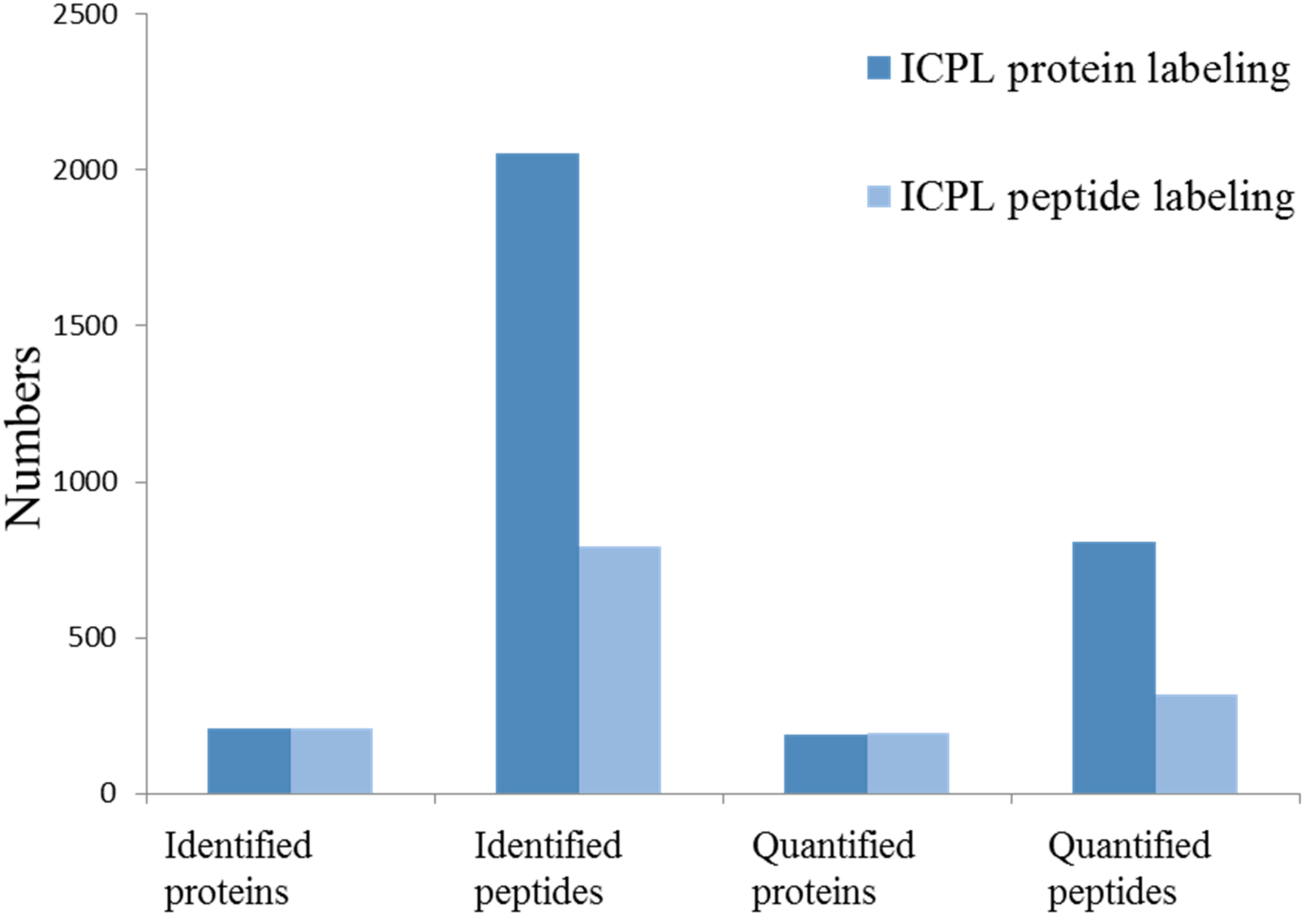
Identified and quantified protein/peptide numbers from protein-labeling and peptide-labeling approaches.

From the ICPL peptide labeling strategy using offline SCX nanoLC-MALDI-TOF/TOF analysis, 793 peptides and 209 proteins were identified and 316 peptides and 192 proteins were quantified. From the ICPL protein strategy with SDS-PAGE protein separation and nanoLC-MALDI-TOF/TOF analysis, 2051 peptides and 208 proteins were identified and 807 peptides and 190 proteins were quantified. (Fig 4) Both experiments were performed once. In addition, only 64 proteins were co-detected in the two strategies, indicating the two methods together confer a highly comprehensive approach for quantitative proteomics research. To evaluate the quantification reproducibility between the two strategies, the quantified values of the same protein in the two strategies were compared. We found that 35% of the co-identified proteins had an RSD of less than 10%, 21% of them was in the RSD range of 10%-20%, 38% of them was in the RSD range of 20%-40%, S1 Table shows 92 proteins with significant fold changes (ratio > 1.5) among control, T1DM, and T1DM/AFB1 groups. Among these proteins, 67 of them were identified with the ICPL protein labeling method, 42 of them with the peptide labeling method, and 17 of them in both methods.

### 3.4 Pathway analysis of differentially expressed proteins

**Fig 5:**
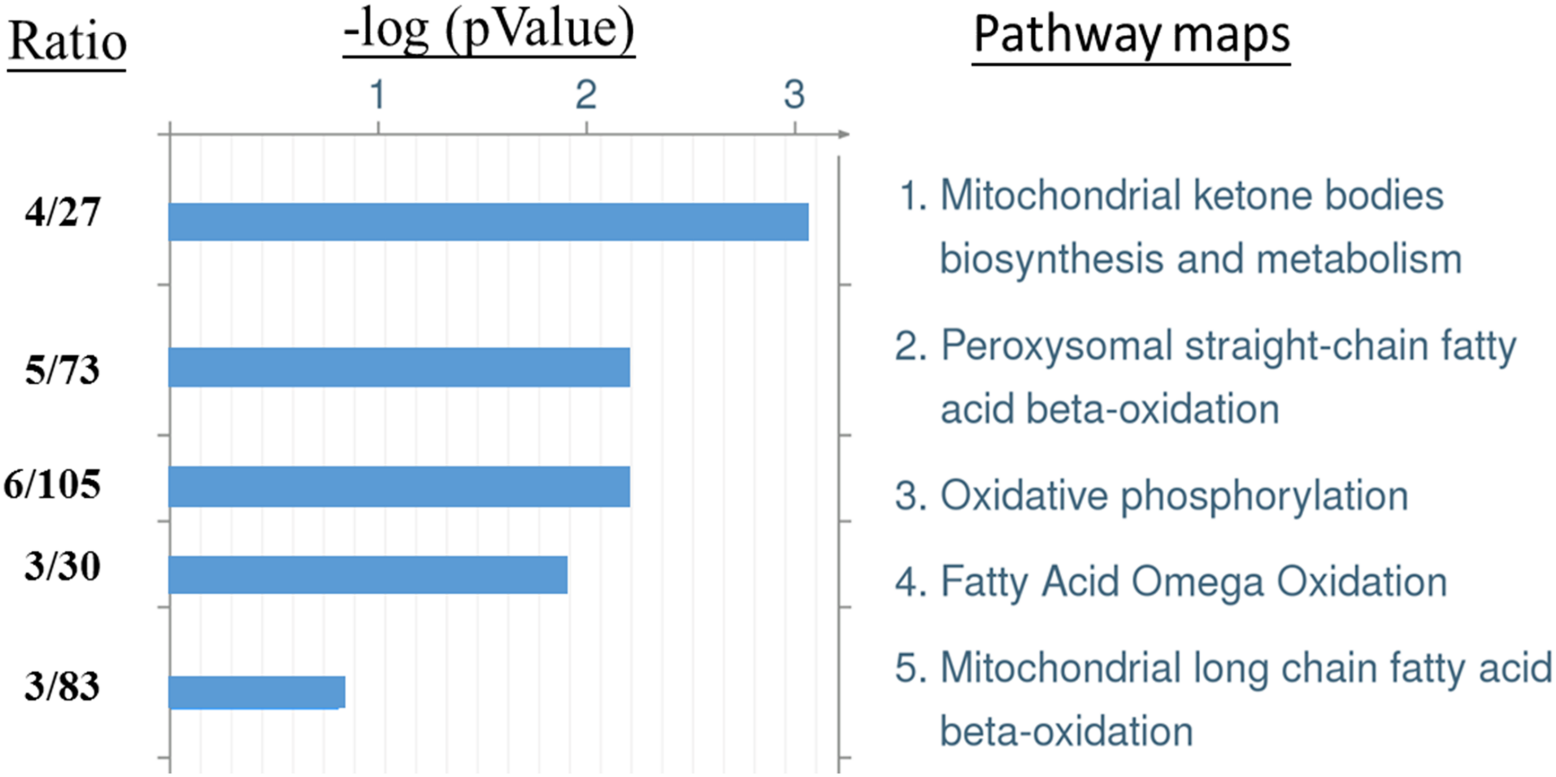
Top 5 significant pathway maps regulated by T1DM and AFB1. The differential-expressed proteins were selected for MetaCore pathway analysis. The statistical tools (-log *P*, *P* value) evaluate relative connectivity of proteins of different functions. The ratio indicates the number of matched proteins from the differential expressed proteins to the number of expected proteins of whole database associated with the respective pathway.

The differentially expressed proteins were analyzed with pathway analysis using MetaCore. The top five most statistically significantly pathway maps are shown in Fig 5. These includes mitochondrial ketone bodies biosynthesis and metabolism, peroxysomal straight-chain fatty acid beta-oxidation, oxidative phosphorylation, fatty acid omega oxidation and mitochondrial long chain fatty acid beta-oxidation. The proteins involved in each pathway were listed in S2 Table.

## 4. Discussion

### 4.1 ICPL proteomics

In 2D-PAGE, with the restricted detection limit of conventional staining methods (coomassie blue staining or silver staining), most proteins visualized in 2D gels are high-abundance proteins. Furthermore, very small and very large proteins, alkaline proteins, and hydrophobic proteins are difficult to be analyzed on 2D gels.[24] Therefore, compared to 2D-PAGE proteomics, the 2-dimentional LC-MS-based stable isotope labeling approaches can uncover more low abundant proteins that play key roles in cellular processes.[25] In addition, because protein isoforms and modifications are separated by 2D-PAGE, the quantification ratios of the multi protein spots on 2D-PAGE should be considered for their quantification results. In contrast, the protein quantification results of ICPL are directly related to detected peptide ratios, which could better represent total expression changes in a given protein.

In nanoLC-MALDI-TOF/TOF platform, the sample/matrix deposition on a MALDI plate can affect the sensitivity of MALDI signals. We found that solvent composition of IPA/ACN/acetone/0.1%TFA (2:7:7:2) was the best CHCA solvent composition to have peptide signal homogeneity, peptide yield and MS/MS durability. This solvent composition was adopted in our nanoLC-MALDI-TOF/TOF platform to investigate the liver proteome.

In our study, the ICPL protein labeling method followed by SDS-PAGE subfractionation provided higher numbers of identified peptides and proteins than the ICPL peptide labeling followed by SCX subfractionation, indicating better peptide recovery from SDS-PAGE. In addition, in comparison to gel-based separation techniques, SCX separation includes time spent both in LC equilibrium and condition optimization. Moreover, multiple desalting steps of SCX-fractioned samples may incur some sample loss.

### 4.2 Mitochondrial ketone bodies

Diabetic ketoacidosis is a potentially lethal complication in DM patients. Ketoacidosis increases the ratio of beta-hydroxybutyrate to oxaloacetate, resulting in depression of the TCA cycle and activation of ketone body production. In our study, beta-hydroxybutyrate dehydrogenase (BDH1) was upregulated in the T1DM group with T1DM/control ratio of 1.45 (S1 Table); BDH1 catalyzes the interconversion of acetoacetate and beta-hydroxybutyrate, which are two ketone bodies produced from fatty acid catabolism. Higher expression of BDH1 in DM may indicate accumulation of beta-hydroxybutyrate, which has been used to diagnose diabetic ketoacidosis [26, 27]. In the T1DM/AFB1 treated group, BDH1 was significantly reduced compared to T1DM group (DM/AFB1 = 1.69). Hydroxymethylglutaryl-CoA lyase was found to be similar between control and T1DM group, but significantly decreased in T1DM/AFB1 group.

### 4.3 Lipid metabolism

In T1DM, the loss of glucose due to insulin deficiency leads to increased dependence upon lipid oxidation for energy metabolism [28], the main mechanism of which is beta oxidation of fatty acids. Fatty acid beta oxidation is a multi-step process wherein fatty acids are broken down to form acyl-CoA, which then enters TCA cycle. In our study, the fatty acid beta oxidation enzymes 17beta-hydroxysteroid dehydrogenase IV and trifunctional enzyme subunit alpha (ECHA) were identified in the ICPL protein and peptide data, and are all increased in T1DM group compared to control group with TIDM/control ratios of 3.03 and 1.59, respectively (S1 Table).

However, expression levels of the two proteins were significantly reduced in T1DM/AFB1 mice (AFB1/DM of 17beta-hydroxysteroid dehydrogenase IV and ECHA = 0.42, 0.63, respectively). In addition, peroxisomal multifunctional enzyme type 2, enoyl-CoA hydratase (ECH), sterol carrier protein 2 and 3-ketoacyl-CoA thiolase A have similar expression level between control and TIDM, but they are significantly reduced in T1DM/AFB1 group. These results may illustrate that lipid metabolism disorder may occur in the T1DM/AFB1 group. Disorder of lipid metabolism in rat liver after AFB1 exposure was also reported by Lu, et al [29].

### 4.4 Oxidative phosphorylation

Johnson et al. [30] have reported that the capacity of the electron transport chain to produce ATP increased in the diabetic liver. Cytochrome c1 (a heme protein)(control/T1DM = 1.14, DM/AFB1 = 1.63), ATP synthase subunit beta (ATP5B) (control/T1DM = 0.92, DM/AFB1 = 1.58), and ATP synthase (H^+^ transport, mitochondrial F1 complex, gamma polypeptide 1) (control/T1DM = 0.78, T1DM/AFB1 = 1.92) and NADH dehydrogenase [ubiquinone] 1 beta subcomplex subunit 10 (control/T1DM = 0.91, T1DM/AFB1 = 1.50) are all involved in oxidative phosphorylation and were found to have similar or slightly decreased expression in the T1DM group as compared to controls. Following treatment with AFB1, levels of these three proteins were significantly decreased and may further illustrate that AFB1 can cause insufficient ATP production in the T1DM group.

### 4.5 Gluconeogenesis and urea cycle

In additional to the major above-mentioned pathways indicated by MetaCore, we also found that several proteins were involved in gluconeogenesis and urea cycle. In type 1 diabetes, loss of insulin secretion leads to an imbalance in glucose metabolic pathways, and blood glucose is regulated by glycogen metabolism through the stimulation of glycogenesis and inhibition of glycolysis.[31]. According to a previous study, gluconeogenesis increases up to 12-fold in rabbits with alloxan-induced diabetes [32]. In our study, the expression level of fructose-bisphosphate aldolase B (aldolase B) in the T1DM group has a ~1.79-fold increase compared to control group. In the T1DM/AFB1 group, aldolase B showed a minor decrease in expression as compared to the T1DM group, which may imply that AFB1 may cause liver cell dysfunctional and impaired gluconeogenesis in TIDM group. Based on transcriptomics and metabonomics results, disorder of gluconeogenesis was also found in rat liver after AFB1 exposure.[29]

The urea cycle, a by-product of protein and amino acid catabolism, takes place primarily in the liver, and diseases related to liver dysfunction are often due to urea cycle disorders. Many of the enzymes in the urea cycle are upregulated in DM [30, 33]. Argininosuccinate synthetase(control/T1DM = 2.74, T1DM/AFB1 = 1.08), argininosuccinate synthase (control/T1DM = 1.73, T1DM/AFB1 = 0.86)) and arginase-1(control/T1DM = 1.68, T1DM/AFB1 = 0.96), participants in the urea cycle, were decreased in the T1DM group in our study. Argininosuccinate synthetase/synthase (ASS) synthesize argininosuccinate by utilizing citrulline. Arginase-1 is the final enzyme of the urea cycle, and catalyzes arginine and water into ornithine and urea. Reduced expression of ASS and arginase-1 in the T1DM group indicate insufficient ammonia metabolism, which can elevate ammonia concentrations in the blood and reach toxic levels (hyperammonemia), which in turn disrupts normal central nervous system function. Because ASS and arginase-1 have similar expression levels across T1DM groups, AFB1 may not influence the urea cycle.

### 4.6 Ribosomal proteins and major urinary proteins

The expression of 60S ribosomal proteins L28 (control/T1DM = 0.93) and L8 (control/T1DM = 0.85) were similar between control and T1DM groups, but were both significantly reduced in the T1DM/AFB1 group, by about 11- and 23-fold, respectively (S1 Table). 40S ribosomal protein S4 (control/T1DM = 1.20, T1DM/AFB1 = 2.63) was also decreased following AFB1 treatment. The ribosome is a complex of ribosomal RNA and ribosomal proteins that catalyze protein translation. Therefore, it is expected that the toxicity and carcinogenicity of AFB1 could lead to inhibition of nuclear transcription and cytoplasmic translation in various hepatic systems.

**Fig 6:**
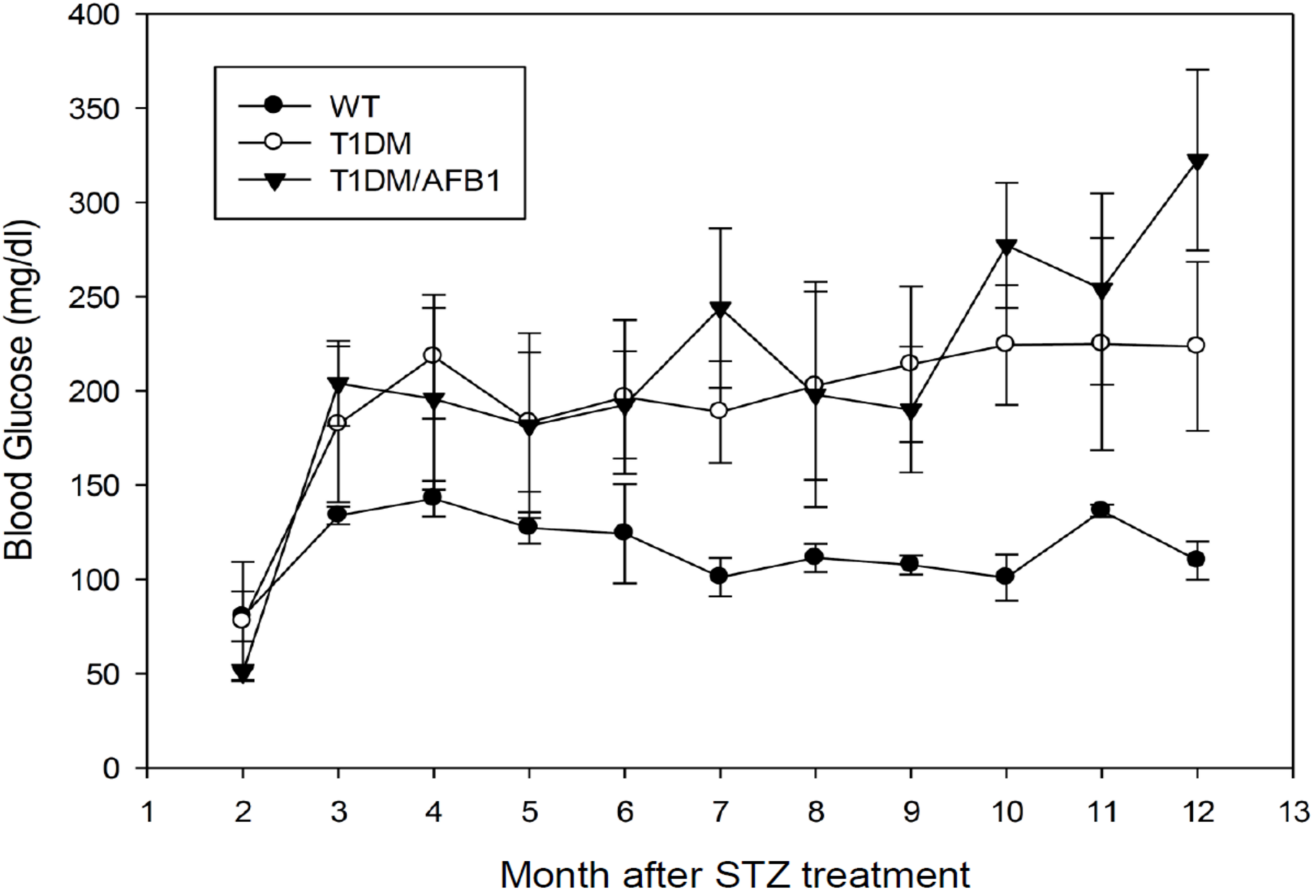
Blood glucose level monitoring in animal models. WT (n = 6) (●), T1DM (n = 6) (○) and T1DM/AFB1 (n = 4) (▼) following buffer alone or STZ treatment.

We also noted that while major urinary protein 1 (MUP1) (control/T1DM = 1.18, T1DM/AFB1 = 5.23), MUP2 (control/T1DM = 0.97, T1DM/AFB1 = 4.53), and MUP11/MUP8 (control/T1DM = 0.97, T1DM/AFB1 = 6.17) were also found to have similar expression levels in the control and T1DM groups, they were significantly decreased in the T1DM/AFB1 group. (S1 Table) MUP belongs to the lipocalin family and is abundantly secreted into circulation by the liver. Overexpression of MUP1 can lower blood glucose levels and cause glucose intolerance with enhanced insulin sensitivity [34]. Recently, MUP1 was found to have decreased expression level in urinary exosomes of diabetic nephropathy [35]. As such, MUP1 is considered a systemic glucose and lipid metabolism regulator in the liver. Fig 6. shows that glucose levels in the T1DM and T1DM/AFB1 groups were higher than those of the control group. Of note, in the latter period (10–12 months), the glucose level of the T1DM/AFB1 group is higher than in the T1DM group, and may indicate worsening insulin sensitivity in the T1DM/AFB1 group as compared to the T1DM group. This phenomenon indicates that AFB1 may decrease the expression of MUP in the liver, resulting in elevated blood glucose due to reduced insulin sensitivity.

## 5. Conclusions

In this study, we have found that CHCA solvent composition of IPA/ACN/acetone/0.1%TFA (2:7:7:2) can efficiently concentrate nanoLC elutes on an anchor chip for MALDI-TOF/TOF analysis. This nanoLC-MALDI-TOF/TOF was applied to ICPL quantitative proteomics for revealing the possible effects of AFB1 on T1DM mice. The novel proteomics findings showed that gluconeogenesis, lipid, and oxidative phosphorylation were elevated in T1DM mice, but these mechanisms were disordered following AFB1 treatment. MUP, which can increase insulin sensitivity, was significantly decreased in the T1DM/AFB1 group, and may results in higher blood glucose levels than in the T1DM group.

## Supporting information

S1 Table Identified proteins with fold changes ≧ 1.5 among control, T1DM and T1DM/AFB1 groups by ICPL-LC-MALDI-TOF/TOF analysis. (DOCX)

S2 Table Differential-expressed proteins involved in top 5 pathway maps. (DOCX)
