# Supplementary material for: The comparison of CHCA solvent compositions for improving LC-MALDI performance and its application to study the impact of aflatoxin B1 on the liver proteome of diabetes mellitus type 1 mice
Source: PLoS One. 2017 Jul 24;12(7):e0181423. doi: 10.1371/journal.pone.0181423 (PMC5524319; doi:10.1371/journal.pone.0181423)
Supplement: S1 Table — (DOCX) [file pone.0181423.s001.docx]

| **S1 Table.** Identified proteins with fold changes ≧ 1.5 among control, T1DM and T1DM/AFB1 groups by ICPL-LC-MALDI-TOF/TOF analysis. | | | | | | | | |
| --- | --- | --- | --- | --- | --- | --- | --- | --- |
| Protein  label | Peptide  label | Protein name | Fold change (N^a^, SD^b^) | | Gi no. | Mw  (kDa) | pI |  |
|  |  |  | Control/DM | DM/AFB1 |  |  |  |  |
|  | √ | 10 kDa heat shock protein | 0.84(1, 0) | 0.51(2, 0.33) | 6680309 | 11.06 | 9.06 |  |
|  | √ | 17beta-hydroxysteroid dehydrogenase IV | 0.33 (2, 0.22) | 2.36 (2, 0.28) | 1213008 | 80.09 | 9.44 |  |
| √ | √ | 3-ketoacyl-CoA thiolase A | 0.92(4, 0.06) | 2.08(2, 0.34) | 18700004 | 46.06 | 9.74 |  |
| √ | √ | 3-ketoacyl-CoA thiolase B | 0.77 (2, 0.05) | 2.99(1, 0) | 22122797 | 46.27 | 9.83 |  |
| √ |  | 40S ribosomal protein S4 | 1.20(1, 0) | 2.63(1, 0) | 4506725 | 32.64 | 10.83 |  |
| √ | √ | 4-hydroxyphenylpyruvate dioxygenase | 1.32(1, 0) | 0.61(1, 0) | 849053 | 48.33 | 6.84 |  |
| √ |  | 60S ribosomal protein 28 | 0.93(2, 0.04) | 11.07(1, 0) | 4506621 | 17.25 | 12.60 |  |
| √ |  | 60S ribosomal protein L26 | 0.89(2, 0.04) | 6.91(1,0) | 4506621 | 16.13 | 10.99 |  |
| √ |  | 60S ribosomal protein L7a | 1.95(1, 0) | N.Q. | 7305443 | 34.22 | 11.19 |  |
| √ |  | 60S ribosomal protein L8 | 0.85(3, 0.02) | 23.29(1, 0) | 4506663 | 30.97 | 11.86 |  |
| √ |  | Adenylate kinase isozyme 2 | 0.83(2, 0.01) | 1.79(2, 0.11) | 4760598 | 27.93 | 7.69 |  |
| √ | √ | ADP/ATP translocase 2 | 0.87(1, 0) | 1.69 (1, 0) | 22094075 | 35.66 | 10.26 |  |
|  | √ | Alpha-1-globin | 1.01(6, 0.05) | 1.81(6, 0.13) | 553919 | 13.06 | 6.96 |  |
| √ |  | Alpha-actinin-4 | 0.84(6, 0.05) | 1.64 (3, 0.16) | 11230802 | 111.25 | 5.13 |  |
| √ |  | Arginase-1 | 1.68(5, 0.06) | 0.96(4, 0.02) | 7106255 | 37.69 | 6.58 |  |
| √ | √ | Argininosuccinate synthase (ASS) | 1.73(17, 0.05) | 0.86(17, 0.04) | 6996911 | 50.31 | 9.04 |  |
| √ | √ | Argininosuccinate synthetase (ASS) | 2.74(2, 0.01) | 1.08(2, 0.01) | 192065 | 22.77 | 8.79 |  |
| √ |  | ATP synthase subunit O, mitochondrial precursor | 1.23(3, 0.08) | 1.85(3, 0.08) | 20070412 | 25.61 | 10.49 |  |
| √ |  | ATP synthase, H+ transporting, mitochondrial F1 complex, gamma polypeptide 1 | 0.78(8, 0.07) | 1.92(9, 0.09) | 122889680 | 18.91 | 10.39 |  |
| √ | √ | ATP synthase subunit beta, mitochondrial (ATP5B) | 0.92(2, 0.03) | 1.58(2, 0.02) | 23272966 | 59.05 | 5.12 |  |
| √ |  | Beta-hydroxybutyrate dehydrogenase (BDH1) | 0.69(3, 0.04) | 1.69(4, 0.16) | 20071589 | 30.82 | 9.11 |  |
| √ |  | Beta globin | 1.06(2, 0.01) | 3.36(2, 0.04) | 193775 | 2.77 | 5.24 |  |
| √ | √ | Betaine--homocysteine S-methyltransferase 1 | 1.87(3, 0.2) | 0.46(4, 0.06) | 7709990 | 48.60 | 8.95 |  |
|  | √ | Beta-lactamase-like protein 2 | 0.56(2, 0.01) | 2.01(3, 0.38) | 21703764 | 33.12 | 5.88 |  |
| √ |  | Bifunctional ATP-dependent dihydroxyacetone kinase/FAD-AMP lyase | 0.74(1, 0) | 1.52(2, 0.12) | 21703976 | 62.88 | 6.48 |  |
| √ |  | Calcium-binding mitochondrial carrier protein Aralar2 isoform 1 | 0.88(2, 0.02) | 1.68(3, 0.02) | 7657583 | 79.23 | 9.41 |  |
| √ |  | Calreticulin precursor | 1.04(1, 0) | 1.66(1, 0) | 6680836 | 52.65 | 4.18 |  |
| √ | √ | Carbonic anhydrase 3 | 1.3(3, 0.03) | 3.54(3, 0.47) | 31982861 | 31.63 | 7.08 |  |
|  | √ | Carbonic anhydrase III | 1.41(1, 0) | 2.02(2, 0.55) | 226778 | 29.72 | 8.90 |  |
| √ | √ | Chain A, 1.8 Angstroms Molecular Structure Of Mouse Liver Class Pi Glutathione S-Transferase Complexed With S-(P-Nitrobenzyl) Glutathione And Other Inhibitors | 1.25(6, 0.04) | 1.88(7, 0.09) | 576133 | 24.89 | 8.92 |  |
| √ |  | Cytochrome c1, heme protein | 1.14 (2, 0.02) | 1.63 (4, 0.13) | 13385006 | 37.00 | 9.76 |  |
|  | √ | D-dopachrome decarboxylase | 1.19(2, 0.04) | 0.69(2, 0.08) | 3122022 | 13.28 | 6.13 |  |
|  | √ | Echs1 protein | 1.26(3, 0.05) | 0.56(3, 0.05) | 12805413 | 31.74 | 9.72 |  |
| √ |  | Endoplasmin | 1.02(9, 0.03) | 1.76(8, 0.07) | 6755863 | 100.68 | 4.59 |  |
| √ |  | Es31 protein | 1.50(4, 0.17) | 4.77(3, 0.18) | 29476863 | 66.52 | 5.74 |  |
|  | √ | Fructose-bisphosphate aldolase B (aldolase B) | 0.56 (13, 0.04) | 1.29(4, 0.1) | 15723268 | 40.11 | 9.40 |  |
| √ |  | Ferrochelatase, isoform CRA_a | 1.37(2, 0.06) | 2.67(2, 0.13) | 148677758 | 17.27 | 11.83 |  |
|  | √ | Fumarate hydratase 1 | 0.82(2, 0.8) | 0.52(1, 0) | 13543801 | 54.67 | 9.72 |  |
| √ | √ | Glutathione S-transferase Mu 1 | 0.94(7, 0.02) | 0.61(7, 0.04) | 6754084 | 26.17 | 8.71 |  |
| √ | √ | Glycine N-methyltransferase | 1.78(3, 0.12) | 0.54(2, 0.01) | 6754026 | 33.22 | 7.81 |  |
|  | √ | Hemoglobin beta | 0.93(7, 0.02) | 1.65(6, 0.03) | 229255 | 15.73 | 8.03 |  |
| √ | √ | Heterogenous nuclear ribonucleoprotein A2/B1 | 0.92(4, 0.05) | 2.23(4, 0.39) | 3329498 | 36.13 | 9.10 |  |
|  | √ | Histone H2A.Z | 0.78(2, 0.06) | 2.20(2, 0.11) | 4504255 | 13.65 | 11.05 |  |
|  | √ | Histone H2B type 3-A | 0.45(2, 0.26) | 3.06(2, 0.98) | 13386452 | 14.09 | 10.80 |  |
|  | √ | HMG CoA synthase | 0.53(1, 0) | 0.62(2, 0.01) | 555837 | 15.81 | 9.68 |  |
| √ |  | IgM B-cell receptor associated protein (BAP) 37 | 0.96(4, 0.06) | 1.87(4, 0.01) | 541734 | 35.25 | 10.21 |  |
| √ |  | Indolethylamine N-methyltransferase | 1.32(2, 0.01) | 1.86(2, 0.01) | 6678281 | 31.85 | 5.99 |  |
| √ |  | Isocitrate dehydrogenase 1 (NADP^+^) | 1.15(3, 0.01) | 1.61(3, 0.12) | 57242927 | 51.03 | 6.88 |  |
| √ |  | Lamin A | 0.62(2, 0.04) | 1.97(2, 0.11) | 220474 | 49.89 | 6.66 |  |
| √ |  | Lamin B2 | 1.27(2, 0.01) | 2.07(2, 0.01) | 52867 | 70.67 | 5.33 |  |
| √ |  | Major urinary protein 1 (MUP1) | 1.18(5, 0.11) | 5.23(4, 0.16) | 257153315 | 22.03 | 4.86 |  |
|  | √ | mCG114098, isoform CRA_b | 1.05(2, 0.11) | 0.56(2, 0.1) | 148668613 | 29.32 | 9.13 |  |
|  | √ | mCG128607 | 1.06(2, 0.11) | 0.55(2, 0.1) | 148666873 | 45.24 | 8.76 |  |
| √ |  | NADH dehydrogenase [ubiquinone] 1 beta subcomplex subunit 10 | 0.91 (1, 0) | 1.50(1, 0) | 58037109 | 22.66 | 9.08 |  |
| √ |  | Nonmuscle heavy chain myosin II-A | 0.71(6, 0.07) | 1.74(5, 0.16) | 17978023 | 248.94 | 5.42 |  |
|  | √ | Olfactory UDP glucuronosyltransferase | 0.16(2, 0.69) | 1.32(1, 0) | 10441350 | 60.46 | 9.61 |  |
| √ |  | Ornithine aminotransferase, mitochondrial precursor | N.Q. | 0.36(6, 0.02) | 8393866 | 51.45 | 6.20 |  |
| √ | √ | Peroxisomal acyl-CoA oxidase | 1.12(2, 0.13) | 1.93(3, 0.16) | 6429156 | 79.20 | 9.23 |  |
| √ |  | Peroxisomal multifunctional enzyme type 2 | 0.86(2, 0.03) | 1.68(3, 0.05) | 31982273 | 85.09 | 9.44 |  |
| √ |  | Phospholysine phosphohistidine inorganic pyrophosphate phosphatase | N.Q. | 0.56(2, 0.04) | 70778744 | 30.83 | 4.84 |  |
| √ |  | Prohibitin | 0.74(4, 0.05) | 1.88(3, 0.08) | 56206786 | 23.99 | 5.96 |  |
| √ |  | Protein 40kD | 1.14(2, 0.02) | 1.63(2, 0.03) | 226005 | 34.00 | 4.65 |  |
| √ | √ | Catalase | 0.94(14, 0.02) | 1.77(14, 0.03) | 115704 | 63.37 | 8.54 |  |
| √ |  | Dimethylaniline monooxygenase | 1.7(1, 0) | 1.0(1, 0) | 3334185 | 64.55 | 9.63 |  |
| √ |  | Hydroxymethylglutaryl-CoA lyase, mitochondrial | 0.9(2, 0.03) | 1.73(2, 0.1) | 585257 | 36.70 | 9.52 |  |
| √ |  | Cytosolic NADP-isocitrate dehydrogenase | 1.15(2, 0.03) | 1.61(2, 0.1) | 6647554 | 50.92 | 6.54 |  |
| √ | √ | Major urinary protein 2 (MUP2) | 0.97(6, 0.03) | 4.53(6, 0.34) | 127527 | 22.20 | 4.88 |  |
| √ |  | Major urinary proteins 11 and 8 (MUP11/MUP18) | 0.97(5, 0.04) | 6.17(5, 0.84) | 127531 | 18.66 | 4.67 |  |
| √ | √ | Enoyl-CoA hydratase (ECH) | 0.81(6, 0.04) | 1.88(6, 0.14) | 17366737 | 83.39 | 9.87 |  |
| √ |  | Heat shock 70 kDa protein 9 | 1.69(8, 0.59) | 1.03(5, 0.07) | 14917005 | 79.33 | 5.85 |  |
| √ |  | Ribosomal protein L12 | 1.10(1, 0) | 1.89(2, 0.06) | 398048 | 19.53 | 10.26 |  |
| √ | √ | S-adenosyl-L-homocysteine hydrolase | 1.53(2, 0.14) | 0.64(2, 0.03) | 904132 | 48.26 | 5.97 |  |
| √ | √ | S-adenosylmethionine synthase isoform type-1 | 1.27(2, 0.01) | 0.51(5, 0.04) | 19526790 | 46.57 | 5.44 |  |
|  | √ | Selenium-binding liver protein | 1.63(2, 0.19) | 0.52(2, 0.01) | 200952 | 52.99 | 5.96 |  |
| √ |  | Selenium-binding protein 2 | 1.97(2, 0.01) | 0.78(2, 0.01) | 9507079 | 55.90 | 5.75 |  |
| √ |  | Serine/arginine-rich splicing factor 1 isoform 1 | 0.85(5, 0.06) | 2.13(5, 0.12) | 5902076 | 28.68 | 10.83 |  |
|  | √ | Spectrin alpha chain, brain isoform 1 | 0.54(5, 0.1) | 1.30(5, 0.22) | 115496850 | 286.13 | 5.08 |  |
| √ |  | Sterol carrier protein 2, liver | 1.33(3, 0.11) | 2.18(3, 0.23) | 123858454 | 28.02 | 6.10 |  |
| √ |  | Trifunctional enzyme subunit alpha, mitochondrial precursor (ECHA) | 0.63(6, 0.03) | 1.59(6, 0.07) | 33859811 | 90.23 | 9.90 |  |
|  | √ | UDP-glucuronosyltransferase 2B4 | 1.03(2, 0.09) | 1.85(2, 0.64) | 22779901 | 60.91 | 9.41 |  |
|  | √ | Unnamed protein product | 0.50(5, 0.11) | 2.71(5, 0.39) | 26348511 | 107.56 | 6.67 |  |
|  | √ | Unnamed protein product | 0.85(6, 0.14) | 2.26(3, 0.58) | 12846616 | 15.93 | 7.97 |  |
| √ |  | Unnamed protein product | 1(3, 0.33) | 2.02(4, 0.1) | 74198639 | 19.51 | 10.26 |  |
| √ |  | Unnamed protein product | 1.36(2, 0.06) | 1.95(2, 0.19) | 12842885 | 65.51 | 8.55 |  |
|  | √ | Unnamed protein product | 0.86(2, 0.12) | 1.65(2, 0.02) | 51304 | 14.76 | 11.70 |  |
| √ |  | Unnamed protein product | 0.75(28, 0.02) | 1.66(29, 0.08) | 74177777 | 58.12 | 5.73 |  |
| √ |  | Unnamed protein product | 0.79(3, 0.07) | 1.75(3, 0.06) | 55291 | 55.96 | 4.94 |  |
|  | √ | Unnamed protein product | 1.22(3, 0.22) | 0.54(2, 0.03) | 74213681 | 56.08 | 6.11 |  |
|  | √ | Unnamed protein product | 1.67(1, 0) | 0.66(2, 0.06) | 74142785 | 32.77 | 6.00 |  |
| √ |  | Unnamed protein product | 1.19(4, 0.05) | 1.60(3, 0.05) | 12841359 | 49.64 | 9.41 |  |
| √ |  | Unnamed protein product | 0.96 (16, 0.01) | 1.56(18, 0.03) | 74211198 | 54.46 | 9.57 |  |
| √ |  | Uricase | 1.14(10, 0.06) | 1.88(8, 0.06) | 6678509 | 37.98 | 9.18 |  |
| N.Q. : No quantifiable data  a: N= numbers of quantitative peptide numbers  b: SD= standard deviation | | | | | | | | |
