# Supplementary material for: The comparison of CHCA solvent compositions for improving LC-MALDI performance and its application to study the impact of aflatoxin B1 on the liver proteome of diabetes mellitus type 1 mice
Source: PLoS One. 2017 Jul 24;12(7):e0181423. doi: 10.1371/journal.pone.0181423 (PMC5524319; doi:10.1371/journal.pone.0181423)
Supplement: S2 Table — (DOCX) [file pone.0181423.s002.docx]

| **S2 Table.** Differential-expressed proteins involved in top 5 pathway maps. | | | | |
| --- | --- | --- | --- | --- |
| Maps | -log (pValue) | pValue | Ratio | Protein Objects |
| Mitochondrial ketone bodies biosynthesis and metabolism | 3.052 | 8.878e-4 | 4/27 | Beta-hydroxybutyrate dehydrogenase (BDH1); HMG CoA synthase; Hydroxymethylglutaryl-CoA lyase, mitochondrial; Trifunctional enzyme subunit alpha, mitochondrial precursor (ECHA) |
| Peroxysomal straight-chain fatty acid beta-oxidation | 2.192 | 6.432e-3 | 5/73 | 17beta-hydroxysteroid dehydrogenase IV; 3-ketoacyl-CoA thiolase A; Peroxisomal multifunctional enzyme type 2; Enoyl-CoA hydratase (ECH); Sterol carrier protein 2, liver |
| Oxidative phosphorylation | 2.183 | 6.568e-3 | 6/105 | ATP synthase, H+ transporting, mitochondrial F1 complex, gamma polypeptide 1; ATP synthase subunit beta, mitochondrial (ATP5B); Cytochrome c1, heme protein; NADH dehydrogenase [ubiquinone] 1 beta subcomplex subunit 10; Unnamed protein product (Gi: 12841359, UQCRC2); Unnamed protein product (Gi: 74211198, ATP5A) |
| Fatty Acid Omega Oxidation | 1.874 | 1.335e-2 | 3/30 | 17beta-hydroxysteroid dehydrogenase IV; Peroxisomal multifunctional enzyme type 2; Enoyl-CoA hydratase (ECH); Sterol carrier protein 2, liver |
| Mitochondrial long chain fatty acid beta-oxidation | 0.773 | 1.685e-1 | 3/83 | Echs1 protein; HMG CoA synthase; Trifunctional enzyme subunit alpha, mitochondrial precursor (ECHA) |
